# Construction of lncRNA-Mediated Competing Endogenous RNA Networks Correlated With T2 Asthma

**DOI:** 10.3389/fgene.2022.872499

**Published:** 2022-04-11

**Authors:** Zihan Wang, Jintao Zhang, Tao Feng, Dong Zhang, Yun Pan, Xiaofei Liu, Jiawei Xu, Xinrui Qiao, Wenjing Cui, Liang Dong

**Affiliations:** ^1^ Department of Respiratory, Shandong Qianfoshan Hospital, Cheeloo College of Medicine, Shandong University, Jinan, China; ^2^ Department of Respiratory Medicine, Shengli Oilfield Central Hospital, Dongying, China; ^3^ Department of Respiratory, Shandong Provincial Qianfoshan Hospital, Shandong University, The First Affiliated Hospital of Shandong First Medical University, Shandong Institute of Respiratory Diseases, Jinan, China

**Keywords:** asthma, airway biopsy, competing endogenous RNA, long non-coding RNA, microRNA, WGCNA

## Abstract

**Background:** Precise classification has been reported as a central challenge in the clinical research on diagnosis and prediction of treatment efficacy in asthma. In this study, the aim was to investigate the underlying competing endogenous RNA network mechanism of asthma, especially T2 asthma, as well as to find more diagnostic biomarkers and effective therapeutic targets.

**Methods:** Multiple sets of T2 asthma airway biopsy transcription profiles were collected, which involved long non-coding RNA (lncRNA), mRNA, and microRNA (miRNA). DIANA-LncBase, targetscan, mirwalk, and miRDB databases were employed to predict interactions between lncRNAs, miRNAs and target mRNAs. To identify mRNAs correlated with T2 asthma, differential expression and network analyses were conducted through weighted gene co-expression network analysis (WGCNA). Subsequently, the expressions of potential biomarkers were examined through qRT-PCR analysis in the T2 asthma coreinteracting cellular factor (IL-13/IL-33) induced experimental model. Lastly, the ceRNA network was confirmed by plasmid transfection and RNAi experiments in a 16HBE cell line.

**Results:** 30 lncRNAs, 22 miRNAs and 202 mRNAs were differentially expressed in airway biopsies from T2 asthma patients. As indicated by the ROC analysis, the lncRNA, PCAT19, had high diagnostic accuracy (AUC >0.9) in distinguishing T2 asthma patients from non-T2 asthma patients and healthy controls. Furthermore, a competing ceRNA network was established, consisting of 13 lncRNAs, 12 miRNAs, as well as eight mRNAs. The reliability of this network was verified by testing several representative interactions in the network.

**Conclusion:** To the best of our knowledge, this study has been the first to establish an lncRNA-mediated ceRNA regulatory network for studying T2 asthma. The findings of this study may elucidate the pathogenesis and help find potential therapeutic targets for T2 asthma. In T2 asthma, *PCAT19*-dominated ceRNA regulation networks may play a critical role, and *PCAT19* may serve as a potential immune-related biomarker for asthma and other respiratory diseases correlated with eosinophilic inflammation.

## Introduction

Bronchial asthma refers to a disease with severe public health implications, Which has affected all age groups worldwide, especially children (Asher et al., 2021). With the increase in prevalence in wide nations, over 400 million people have been estimated to have asthma by 2025, thus imposing a heavy social burden on the Global Strategy for Asthma Management and Prevention, 2021. Asthma can fall into two major groups (T2 asthma and non-T2 asthma); different asthma types may respond to various treatment regimens (Wenzel, 2012). Despite the above insights, asthma patients are still treated according to the one-size-fits-all principle for a long period. Since targeted drugs for asthma were produced, more accurate Th2 typing methods have been urgently required.

LncRNAs are RNA molecules with a length of over 200 bases without protein-coding potential (Schmitt and Chang, 2016). They were originally considered “transcriptional noise,” since not involved in protein coding. In-depth research and increasing evidence have suggested that the above RNAs play an essential role in multiple aspects (e.g., gene replication, transcriptional regulation, as well as protein function) (Caley et al., 2010; Cech and Steitz, 2014; Iyer et al., 2015). With the development of human genome sequencing, numerous transcriptome genes, especially lncRNAs, were reported to contain miRNA binding sites, thus leading to the proposal of the competing endogenous RNA (ceRNA) hypothesis (Salmena et al., 2011). Non-coding RNAs (ncRNAs) (e.g., lncRNAs, pseudogenes and circular RNAs) could serve as a sponge for miRNAs via miRNA response elements to have an indirect effect on the expressions of protein-coding mRNAs (Sanchez-Mejias and Tay, 2015; Bossi and Figueroa-Bossi, 2016). According to the ceRNA hypothesis, the functions of many characteristically unknown lncRNAs are elucidated. However, the position and mechanism of the lncRNA-meditated ceRNA network involved in T2 asthma remains unclear.

This study aimed to find differentially expressed genes of lncRNAs, miRNAs, and mRNAs in T2 asthma through the GEO database assay (GSE67472 and GSE142237). Their interaction relationships were predicted using DIANA-LncBase, targetscan, miRDB, and mirwalk databases. Lastly, a ceRNA regulatory network relating to T2 asthma was established and verified through quantitative real-time polymerase chain reaction (qRT-PCR). Based on the established ceRNA network in T2 asthma, we can gain insights into the etiology of the disease and may find potential diagnostic tools and therapeutic targets for T2 asthma.

## Materials and Methods

### Data Acquisition

**FIGURE 1:**
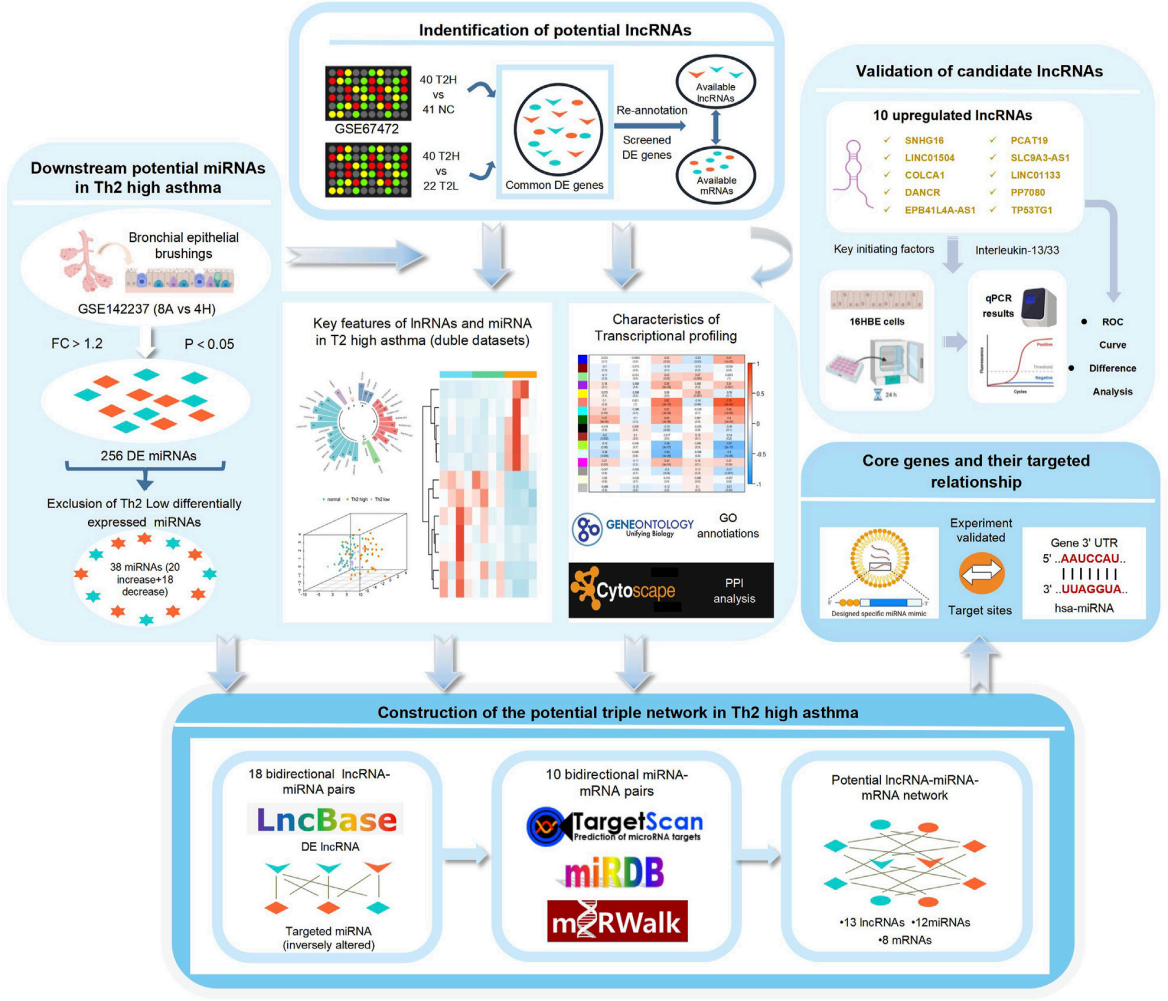
The flow chart of the study.

According to the National Center for Biotechnology Information (NCBI) Gene Expression Omnibus (GEO) database (http://www.ncbi.nlm.nih.gov/geo), all datasets regarding asthma were searched, but only GSE67472 and GSE142237 containing T2 and non-T2 asthma samples were available for further study. The GSE67472 data included genome expression profiling of airway epithelial cells from 62 asthma patients (40 T2 asthma patients and 22 non-T2 asthma patients) and normal bronchial brushings from 43 healthy controls. The GPL16311 Affymetrix Human Genome U133 Plus 2.0 Array was used to analyze the above data. The GSE142237 data, which formed the miRNA dataset, were collected from bronchial biopsy samples in a study conducted at the Huazhong University of Science and Technology in China. The study consisted of 12 cases, including eight asthma cases (4 T2 asthma and four non-T2 asthma cases) and four control cases. Figure 1 illustrates the workflow and data preprocessing.

### Identification of Long Non-Coding RNA and Differential Expression Analysis

The lncRNAs were defined and annotated in accordance with the Gencode lncRNA annotation file (gencode.v31.long_noncoding_RNAs.gtf.gz) from the GENCODE database (https://www.gencode-genes.org). Differentially expressed (DE)mRNAs, DElnRNAs, and DEmiRNAs were acquired from the two datasets using the online analytic tool “GEO2R” (https://www.ncbi.nlm.nih.gov/geo/geo2r), an algorithm based on the R language Linear Models for Microarray data (limma) package. | Fold change | (FC) >1.2 and *p*-value <0.05 were selected as thresholds in differential expression analyses. Furthermore, a Venn diagram was generated to illustrate the intersection of different groups.

### Principal Component Analysis

PCA, conducted using the “scatterplot3d” package of the R software (version 3.6.2), was employed to study the classification ability of the whole lncRNA sets and the overlapping DElncRNAs in patients suffering from T2 asthma and non-T2 asthma and normal individuals.

### Receiver Operating Characteristic Analysis

ROC analysis was performed based on R software’s “pROC” package (version 3.6.2). Area under curve (AUC), defined as the area under the ROC curve, has been commonly adopted to assess models.

### Prediction of the lLong Non-Coding RNA and microRNA Targets

LncRNAs capable of regulating miRNA expression were predicted using the DIANA-LncBase V2 database (https://omictools.com/diana-lncbase-tool) (Paraskevopoulou and Hatzigeorgiou, 2016). mRNA targets of DEmiRNAs were predicted using Targetscan 7.2 (http://www.targetscan.org/vert_72/) (Agarwal et al., 2015), Mirwalk 2.0 (http://mirwalk.umm.uni-heidelberg.de/) (Chen and Wang, 2020), and miRDB (http://mirdb.org/) (Chen and Wang, 2020).

### Weighted Gene Co-Expression Network Analysis and the Identification of Modules

A weighted gene co-expression network analysis (WGCNA) based on the mRNA expression profile was constructed using the WGCNA R package (Langfelder and Horvath, 2008). First, the top 40% of the genes with the highest variance were employed to conduct data analysis, and the unrecognized genes were eliminated with goodSamplesgenes. Subsequently, the co-expression similarity matrix was transformed into adjacency matrix by selecting an appropriate soft-thresholding power (β), obtained using the pickSoftThreshold method. The adjacency matrix was converted into a TOM using a topological overlap matrix (TOM) similarity function. Lastly, dynamic tree cutting was adopted to cut the clustering tree branches to generate modules. Furthermore, gene significance (GS) was obtained to measure the correlation between gene expression and sample traits (e.g., age, gender, disease state and T2 type), and obtained significance (MS) was used to identify the correlations between modules and sample traits.

### Functional and Pathway Enrichment Analyses of the Overlapping mRNAs

Functional analysis was conducted to gain insights into the functions of overlapping mRNAs. Enrichment analyses were conducted using the “ClusterProfiler” R package in accordance with Gene Ontology (GO) terms, including three domains (molecular function (MF), biological process (BP), and cellular component (CC) categories) and Kyoto Encyclopedia of Genes and Genomes (KEGG) (Yu et al., 2012); *p*-value <0.05 had statistical significance.

### Protein–Protein Interaction Network

Overlapping genes were uploaded to the STRING database (https://www.string-db.org/) to explore the correlation within the PPI network. The screening criterion for the network was confidence over 0.4. Subsequently, the output was visualized from the STRING database using the Cytoscape software. Cytohubba, a plugin of Cytoscape, was adopted to find the hub genes of the mRNAs in the PPI networks.

### Construction of the Long Non-Coding RNA–microRNA–mRNA Regulatory Network

The DElnRNAs fell into two groups in accordance with their expression trends. Subsequently, the predicted downstream miRNAs of DElnRNAs intersected with DEmiRNAs of the opposite strand. Next, the target genes of the miRNA generated in the previous step intersected with the DEmRNAs. Lastly, the interrelationships were illustrated based on the Cytoscape software.

### Cell Culture and Treatment

Human bronchial epithelial cell line 16HBE cells were acquired from the Shanghai FuHeng Biology Co., (Shanghai, China). The cells were seeded into six-well plates and grown for 12 h before transfection or stimulation. After being replaced with fresh pre-warmed media, the cells were transfected with plasmid, siRNA, miR mimics, or negative controls (NC) (GenePharma, China) with the use of the transfection reagent, EndoFectin™-Max (GeneCopoeia), in accordance with the manufacturer’s protocol. In experiments requiring stimulation of interleukins, the cells were stimulated with their corresponding reagents for the indicated periods. The recombinant proteins, recombinant mouse IL-33 protein (MCE) and recombinant human IL-13 protein (Abbikine), were purchased as indicated.

### qRT-PCR and Statistical Analysis

RNA was extracted with the use of the RNAfast200 kit (Fastagen, Shanghai, China). The relative gene expression level was obtained using the 2^−ΔΔCt^ method. To normalize the data, GAPDH served as an internal reference for mRNA, and U6 was employed as an internal reference for miRNA. Student’s t test was performed to compare the differences between the control and experimental conditions, and *p*-value <0.05 indicated statistical significance. Table 1 lists the sequences of the primers.

**TABLE 1:** Primers for qRT-PCR.

Symbol	Primer Sequence (5′–3′)
Human β-Actin	Forward: GGA​AAT​CGT​GCG​TGA​CAT​TAA
	Reverse: AGG​AAG​GAA​GGC​TGG​AAG​AG
Human PCAT19	Forward: GCC​CCC​TGT​GTG​GGA​ATA​G
	Reverse: GCA​GGA​CCC​GAC​TAT​GAG​G
Human SLC9A3-AS1	Forward: GCC​TGT​CCT​GTT​GGA​TGG​AG
	Reverse: TTC​CGT​TAG​GCG​TGT​CCT​G
Human LINC01133	Forward:AGAGCCATGGTACTGGAGGA
	Reverse: TGC​TGG​GCT​CTG​GAT​TCT​TG
Human PP7080	Forward: TTG​ACT​GGT​CGG​TCT​TCA​GC
	Reverse: AGC​CTG​TGC​AAT​TCT​CGT​CA
Human TP53TG1	Forward: CTC​TAT​TCT​GGG​CTG​CGG​G
	Reverse: GAG​GGT​TGG​GTA​CCT​TCG​TG
Human SNHG16	Forward: AGA​CTG​TGC​AAA​GCC​GTG​TA
	Reverse: TGA​CGG​TAG​TTT​CCC​AAG​TTT
Human LINC01504	Forward: CGC​CTG​ACC​CAC​CCT​TAT​TCT​AA
	Reverse: CCG​CAG​CTC​TGG​ATT​TAC​TTC​T
Human COLCA1	Forward: AGG​AGA​AGC​AGC​CGA​GTT​AC
	Reverse: AGC​CGG​ATG​CTT​TGT​GAA​AT
Human DANCR	Forward: GCC​CTT​GCC​CAG​AGT​CTT​C
	Reverse: GCC​CGA​AAC​CCG​CTA​CAT​A
Human EPB41L4A-AS1	Forward: AAA​GGT​GAC​CTG​AAG​GAT​GTC
	Reverse: ATC​ACT​TAA​AAC​ACA​AAT​GCC​AAA​G
Human LBH	Forward: CCATTCACTGCCCCGACT
	GCA​GCA​GCG​GTC​AAA​ATC​TG
Human MAP2K6	Forward: CCA​GGA​ACA​GAA​ACG​GCT​ACT
	Reverse: ACA​TCA​CCC​TCC​CGA​AAC​AG
Human SLC39A10	Forward: AAC​TTC​AGT​GAT​GGG​CTC​GC
	Reverse: CAT​CAT​GGC​AGA​GAG​GAG​GTT
Human POSTN	AGC​AAA​CCA​CCT​TCA​CGG​AT
	ACA​GGT​GCC​AGC​AAA​GTG​TA
hsa-miR-31-5p	Forward: GTG​TTG​TTC​TAA​AGG​CAA​GAT​GC
	Reverse: TAT​GGT​TGT​TCT​CGT​CTC​TGT​GTC
hsa-miR-378d	Forward: ATC​TAT​GCT​CGC​ACT​GGA​CTT​G
	Reverse: TAT​GGT​TGT​TCA​CGA​CTC​CTT​CAC
U6	Forward: CAG​CAC​ATA​TAC​TAA​AAT​TGG​AAC​G
	Reverse: ACG​AAT​TTG​CGT​GTC​ATC​C

## Results

### Identification of DElncRNAs

**FIGURE 2:**
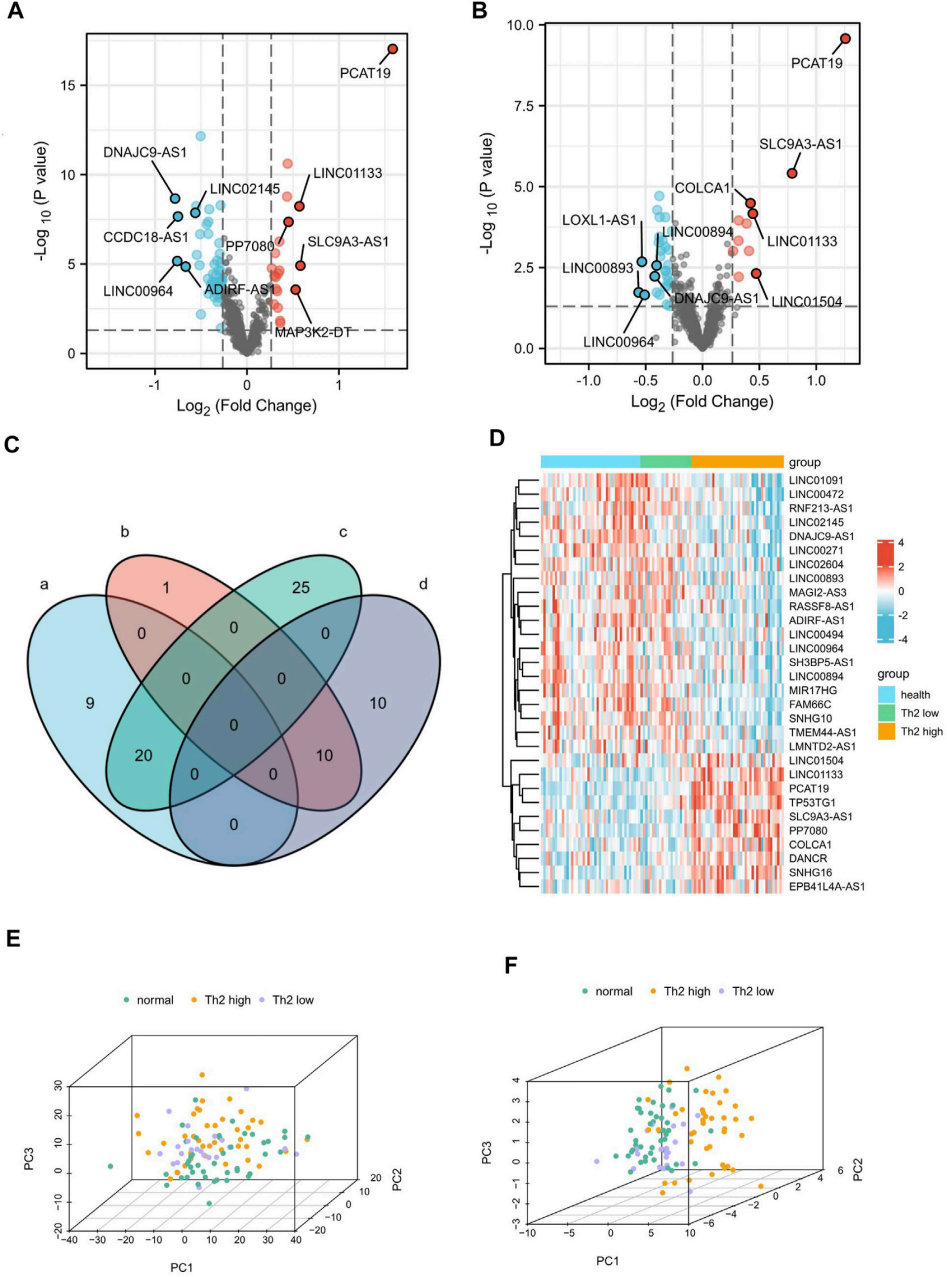
Identification of 30 DElncRNAs related to Th2 high asthma.**(A,B)** Volcano plot of the differentially expressed lncRNAs between patients with Th2 high asthma and healthy controls and between patients with Th2 high asthma and Th2 low asthma. The upregulated and downregulated genes with top five ｜logFC｜were marked. **(C)** A Venn diagram was plotted to identify the upregulated and downregulated DElncRNAs related to Th2 high asthma. **(D)** The heat map of the expression of the 30 DElncRNAs. **(E,F)** Principal component analyses of Th2 high asthma, Th2 low asthma, and healthy control groups based on the whole lncRNA and the 30 overlapping DElncRNAs.

With the above thresholds (fold change ≥1.2 and *p*-value <0.05), 65 DElncRNAs (20 up-regulated and 45 down-regulated) were detected as differentially expressed in T2 asthma patients, compared with healthy controls. Forty DElncRNAs (11 up-regulated and 29 down-regulated) were identified as differentially expressed in T2 asthma patients, compared with non-T2 asthma patients. The volcano plots visualized the variation of lncRNA between T2 asthma patients and controls (Figure 2A) and patients suffering from T2 asthma and non-T2 asthma (Figure 2B).

**FIGURE 3:**
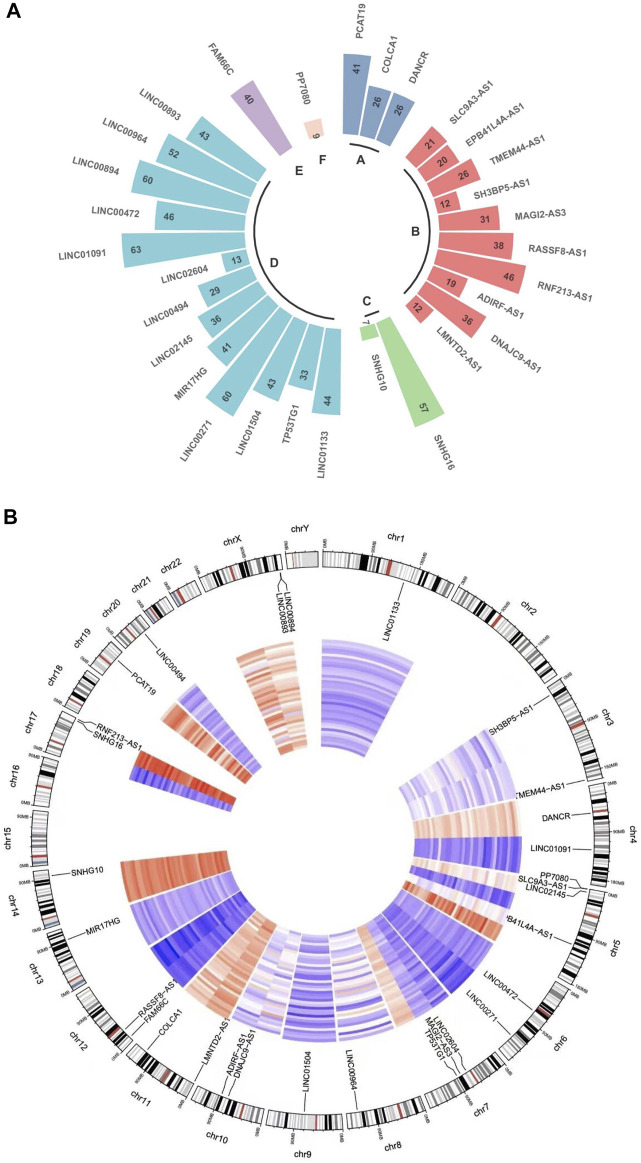
LncRNA function and distribution in every chromosome. **(A)** Circular plot indicates the lncRNA counts and types according to the differentially regulated lncRNA categorizations. The RNA lengths plotted in Log (1.1 kbp) scale. **(A)**: Long non-coding RNAs with non-systematic symbols. **(B)**: Antisense RNAs; **(C)**: Small nucleolar RNA non-coding host genes; **(D)**: Long intergenic non-protein coding RNAs; **(E)**: Long non-coding RNAs with FAM root symbol; **(F)**: Non-specified. (B) A circos diagram showing the location of the identified lncRNA pairs in the chromosome.

The Venn diagram depicts the shared 10 up-regulated (*PCAT19*, *SLC9A3-AS1*, *LINC01133*, *PP7080*, *TP53TG1*, *SNHG16*, *LINC01504*, *COLCA1*, *DANCR,* and *EPB41L4A-AS1*) and 20 down-regulated (*LINC00271*, *MIR17HG*, *LINC02145*, *LINC00494*, *TMEM44-AS1*, *LMNTD2-AS1*, *SH3BP5-AS1*, *MAGI2-AS3, LINC02604*, *RASSF8-AS1, LINC01091*, *FAM66C*, *SNHG10, RNF213-AS1*, *LINC00472*, *ADIRF-AS1*, *LINC00894*, *DNAJC9-AS1*, *LINC00964*, and *LINC00893*) DElncRNAs in both T2 asthma patients compared with healthy controls, and T2 asthma patients compared with non-T2 asthma patients (Figure 2C). Additionally, the heat map showed the expression of the above 30 overlapping DElncRNAs in the three sets (Figure 2D). PCA results indicated that when all expression lncRNAs were studied, the components were intertwined with one other. There was no significant difference between the T2 asthma, non-T2 asthma, and control groups (Figure 2E). However, a clear boundary between the three groups, based on the overlapping DElncRNAs for PCA, suggested that the above lncRNAs may play a role in recognizing asthma and health states, especially T2 asthma (Figure 2F). The functions and distributions of screened lncRNAs in every chromosome were shown in Figure 3.

### The Expression of the Up-Regulated DElncRNAs

**FIGURE 4:**
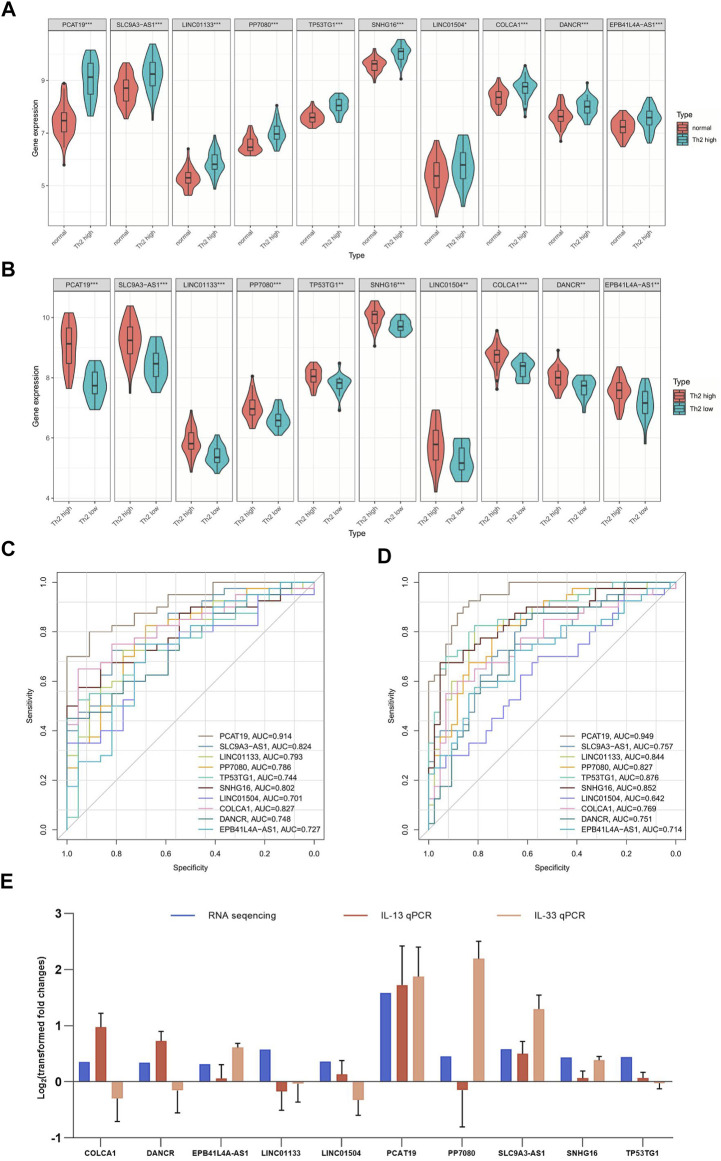
ROC analysis and gene expression of 10 overlapping upregulated lncRNAs. **(A)** ROC analysis of 10 overlapping upregulated lncRNAs between patients with Th2 high asthma and health controls. **(B)** ROC analysis of 10 overlapping upregulated lncRNAs between patients with Th2 high asthma and patients with Th2 low asthma. **(C)** Expression of 10 overlapping upregulated lncRNAs in patients with Th2 high asthma and normal samples. **(D)** Expression of 10 overlapping upregulated lncRNAs in Th2 high asthma and Th2 low asthma. **(E)** The level of identified lncRNAs in IL-13/IL-33-induced 16HBE cells measured by qRT-PCR. *p < 0.05; **p < 0.01.

To further visualize the differential expression of the up-regulated DElncRNAs between T2 asthma and healthy control groups and T2 asthma and non-T2 asthma groups, violin plots (Figures 4A,B) were plotted. The results suggest differences in the above genes between the two groups (P ＜0.05).

### Diagnostic Significance of DElncRNAs

ROC analyses were conducted to verify the sensitivity and specificity of DElncRNAs in the diagnosis of T2 asthma. The up-regulated DElncRNAs were selected for ROC analysis in the groups. The top five AUCs for DElncRNAs of the T2 asthma and healthy control (Figure 4C) groups included *PCAT19* (AUC = 0.949), *TP53TG1* (AUC = 0.876), *SNHG16* (AUC = 0.852), *LINC01133* (AUC = 0.844), and *PP7080* (AUC = 0.827). Similarly, the following results showed that the DElncRNAs with the top five AUCs of the T2 asthma and non-T2 asthma groups (Figure 4D): *PCAT19* (AUC = 0.914), *COLCA1* (AUC = 0.827), *SLC9A3-AS1* (AUC = 0.824), *SNHG16* (AUC = 0.802), and *LINC01133* (AUC = 0.793). Notably, the results suggested that *PCAT19* achieved the best diagnostic value for distinguishing between the T2 asthma patients from those with non-T2 asthma, and it may serve as a promising biomarker for T2 asthma.

As revealed by the characteristic cytokine (IL-13/IL-33)-induced T2 asthma cellular model, the expression trend of only four lncRNAs, EPB41L4A-AS1, PCAT19, SLC9A3-AS1, and SNHG16, was consistent with the results of RNA sequencing with the stimulation of IL-13 and IL-33. The rest lncRNAs were compatible with the RNA sequences under only one stimulus or opposite (Figure 4E).

### Identification of DEmiRNAs

**FIGURE 5:**
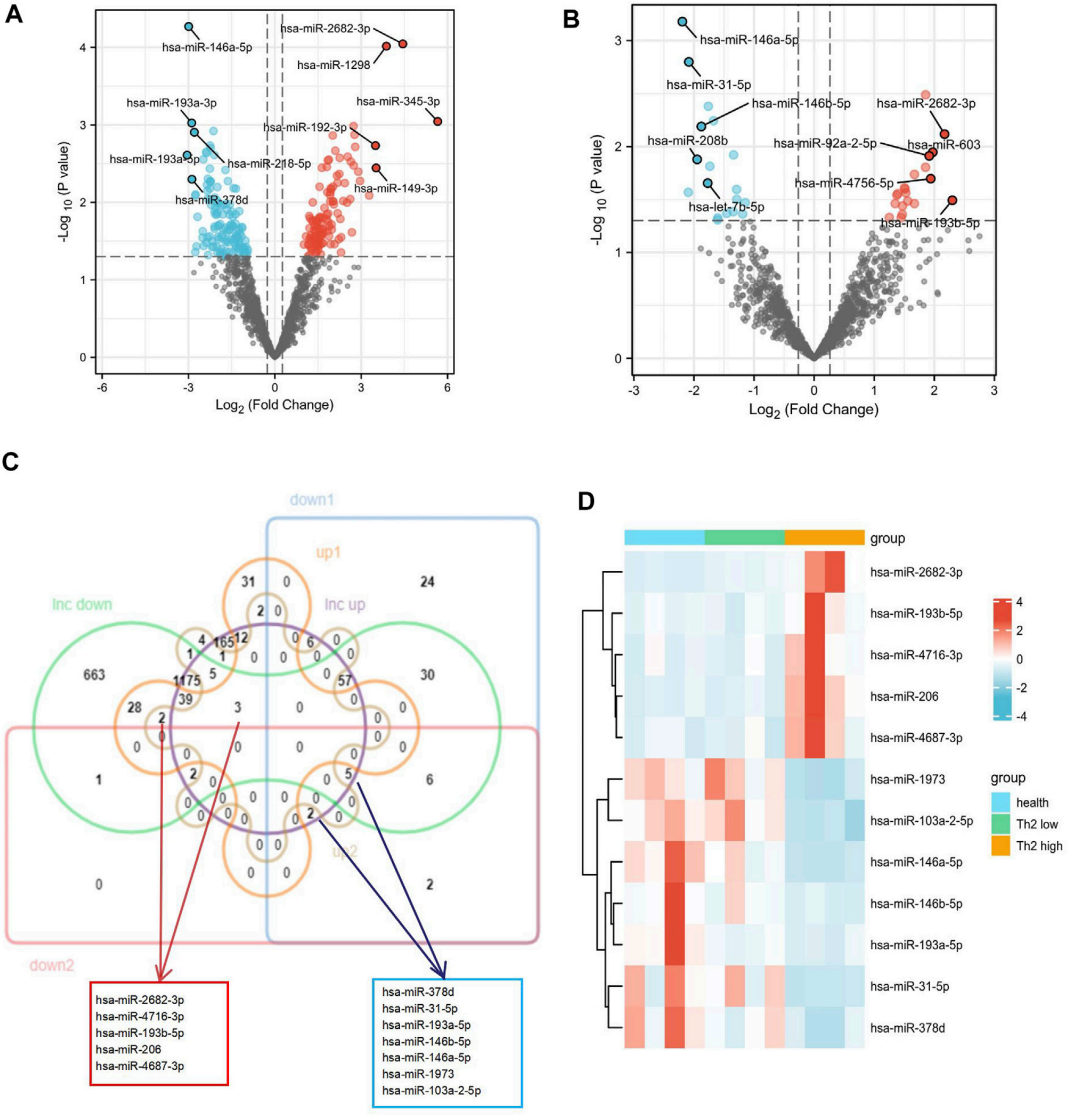
Identification of the DEmiRNAs related to Th2 high asthma. **(A–B)** Volcano plot of the differentially expressed miRNAs between Th2 high asthma and healthy control groups and between Th2 high asthma and Th2 low asthma groups. The upregulated and downregulated genes with top five｜logFC ｜were marked. **(C)** Venn diagram of upregulated and downregulated predicted target DEmiRNAs. **(D)** The heat map of expression of the 12 target miRNAs.

With the above thresholds (fold change ≥1.2 and *p*-value <0.05), 256 DEmiRNAs (124 up-regulated and 132 down-regulated) were detected as differentially expressed in T2 asthma patients, compared with healthy controls. Thirty-eight DEmiRNAs (20 up-regulated and 18 down-regulated) were identified as differentially expressed in T2 asthma patients, compared with non-T2 asthma patients. The volcano plots visualized the variation of miRNA between the T2 asthma and control groups (Figure 5A) and between the T2 asthma and non-T2 asthma groups (Figure 5B). Subsequently, the seven shared up-regulated (*hsa-miR-3146*, *hsa-miR-4716-3p*, *hsa-miR-193b-5p*, *hsa-miR-1252*, *hsa-miR-4687-3p*, *hsa-miR-206*, and *hsa-miR-2682-3p*) and 15 down-regulated (*hsa-miR-193a-3p*, *hsa-miR-103a-2-5p*, *hsa-miR-193a-5p*, *hsa-miR-146a-5p*, *hsa-miR-31-5p*, *hsa-miR-3607-3p*, *hsa-miR-378d*, *hsa-miR-146b-5p*, *hsa-miR-203*, *hsa-miR-208b*, *hsa-let-7b-5p hsa-miR-297*, *hsa-miR-1973*, *hsa-miR-31-3p*, and *hsa-miR-4714-5p*) DEmiRNAs in the comparison between the T2 asthma and health control groups and between T2 asthma and non-T2 asthma groups were obtained. Afterwards, a Venn diagram (Figure 5C) was used to illustrate the intersection between the target miRNAs of 10 up-regulated and 20 down-regulated DElncRNAs, obtained by the above, and the shared up-regulated and down-regulated miRNAs. In addition, the heat map showed the expression of the above 12 overlapping DEmiRNAs in the three sets (Figure 5D).

### Identification of T2 Asthma-Related mRNAs

**FIGURE 6:**
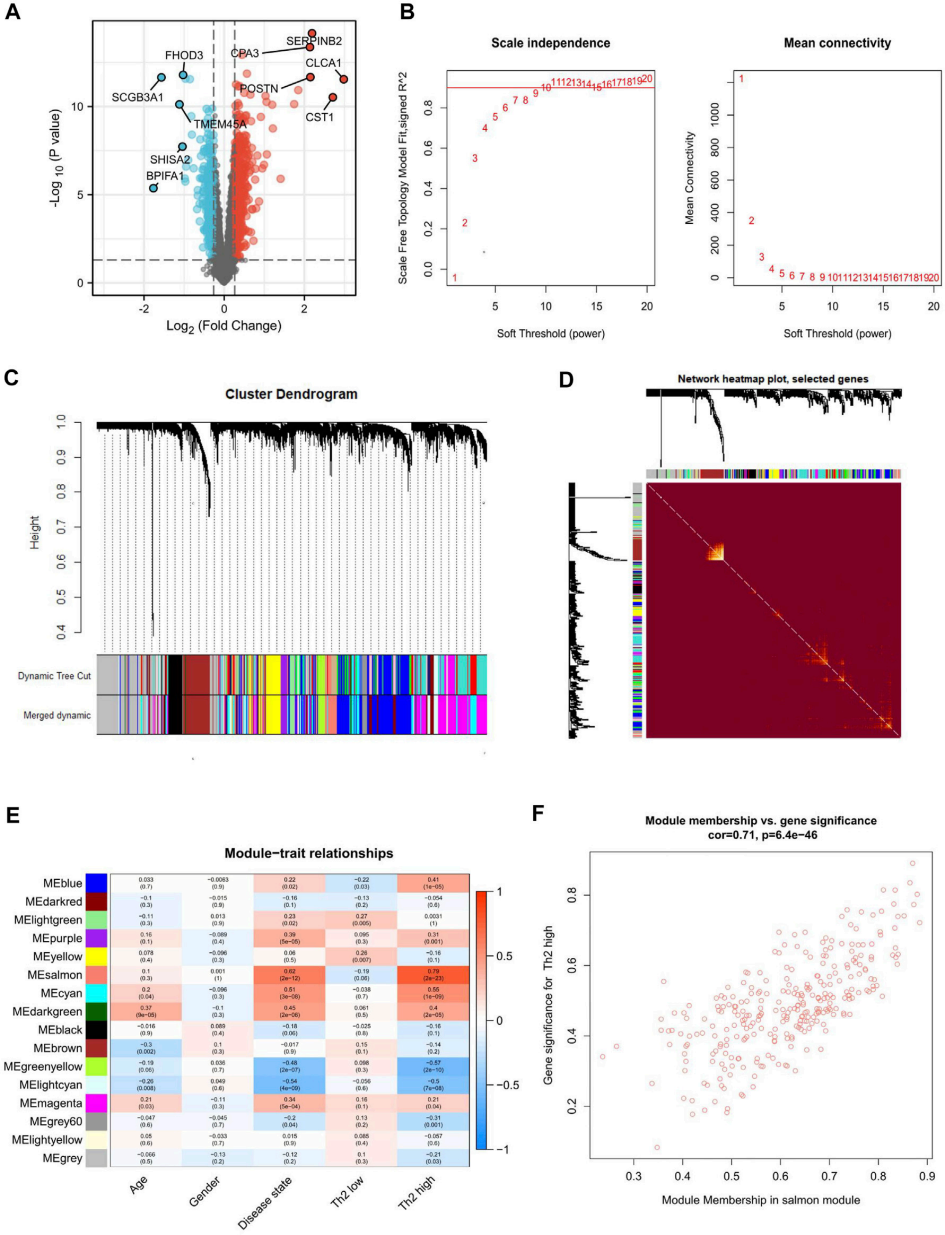
Differential expression analysis and WGCNA identification of Th2 high asthma-associated modules. **(A)** Volcano plot of the differentially expressed mRNAs between asthma and healthy control groups. The upregulated and downregulated genes with top five ｜logFC｜were marked. **(B)** The process of identifying the most appropriate soft threshold. **(C)** Clustering dendrogram of mRNAs with top 40% variances in the co-expression network. **(D)** Heat map depicting the topological overlap matrix (TOM) among genes based on co-expression modules. **(E)** Relationships between mRNA modules and clinical traits. **(F)** Correlation between module membership of salmon module and gene significance with Th2 high asthma.

First, 903 DEmRNAs were identified between the asthma and healthy control groups, with thresholds (fold change ≥1.2 and *p*-value <0.05). The results of the screens were plotted using volcano plots (Figure 6A). Moreover, instead of focusing only on differential gene expression, WGCNA exploits information from the whole mRNA. The first 40% variant mRNAs, measured based on the median absolute deviation (MAD), were selected for the WGCNA. Cluster analysis showed that one study deviated significantly from other studies, which was excluded from the analysis. The soft threshold power was set at 9, with a *R* ^2^ of 0.872 to transform the Pearson’s correlation matrix of the gene into a strengthened adjacency matrix (Figure 6B). 16 gene color modules were detected (Figure 6C); the heat map plot of TOM is visualized in Figure 6D. Subsequently, the correlation between each module and its corresponding clinical traits (age, gender, disease state, and asthma type) is shown in Figure 6E. Among the above 16 modules, the salmon module (*R* = 0.71, *p* < 0.01) had a correlation with T2 asthma (Figure 6F).

### Functional Analysis and Protein–Protein Interaction of the DEmRNAs Related to T2 Asthma

**FIGURE 7:**
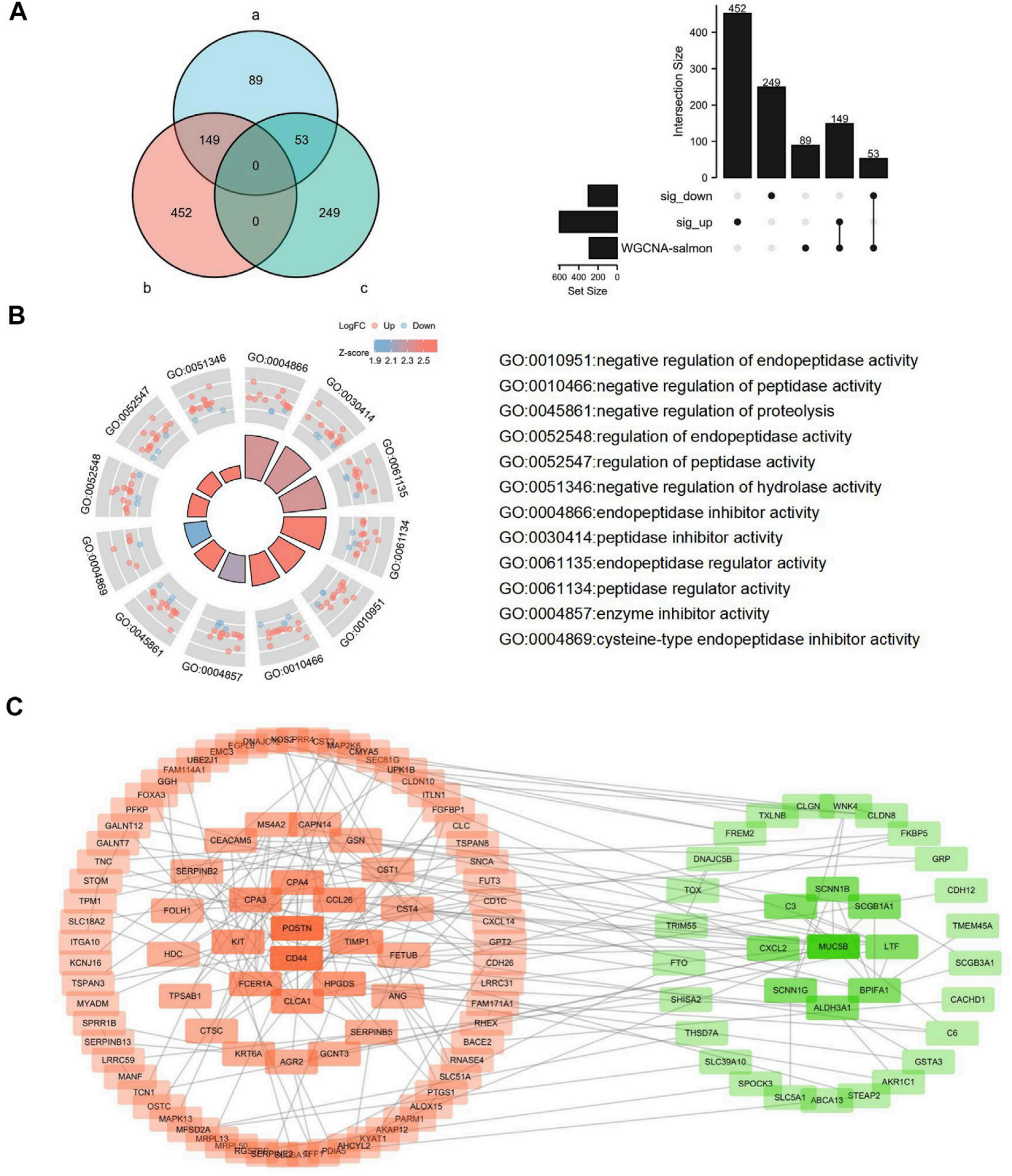
Identification, functional analysis, and PPI analysis of the DEmRNAs related to Th2 high asthma. **(A)** Overlapping DEmRNAs were analyzed using the WGCNA-salmon mRNAs, the significantly upregulated DEmRNAs and downregulated DEmRNAs. **(B)** Functional analysis of the overlapping DEmRNAs. **(C)** PPI analysis of the overlapping DEmRNAs.

T2 asthma-related genes in the Salmon module analyzed by WGCNA were intersected with DEmRNAs identified in asthma patients and healthy controls. The results (Figure 7A) showed that 149 up-regulated and 53 down-regulated T2 asthma-related DEmRNAs were present. To explore the function of the above DEmRNAs, the above differential genes were analyzed using the Clusterprofiler R package. The results indicated that the above DEmRNAs mainly elevated negative regulatory activities, such as negative regulation of endopeptidase activity and peptidase activity (Figure 7B).

To explore potential PPIs in T2 asthma, 202 DEmRNAs were uploaded to the STRING database for in-depth analysis (http://string.embl.de/). A median confidence of 0.4 was adopted as the cut-off. 124 nodes and 185 edges were screened after the disconnected nodes were removed in the network. Subsequently, the network was visualized using the Cytoscape software after the disconnected nodes were hided (Figure 7C). Next, the CytoHubba was adopted to find the hub genes of the DEGs. *CD44*, *MUC5B*, *POSTN*, *CLCA1*, *CPA3, BPIFA1*, *CCL26*, *CPA4*, *KIT* and *HPGDS* were ranked using the degree method. ROC analysis was conducted to indicate the diagnostic value on T2 asthma of the hub genes (Supplementary Figure S1).

### Construction and Verification of the Long Non-Coding RNA-microRNA-mRNA ceRNA Network

**FIGURE 8:**
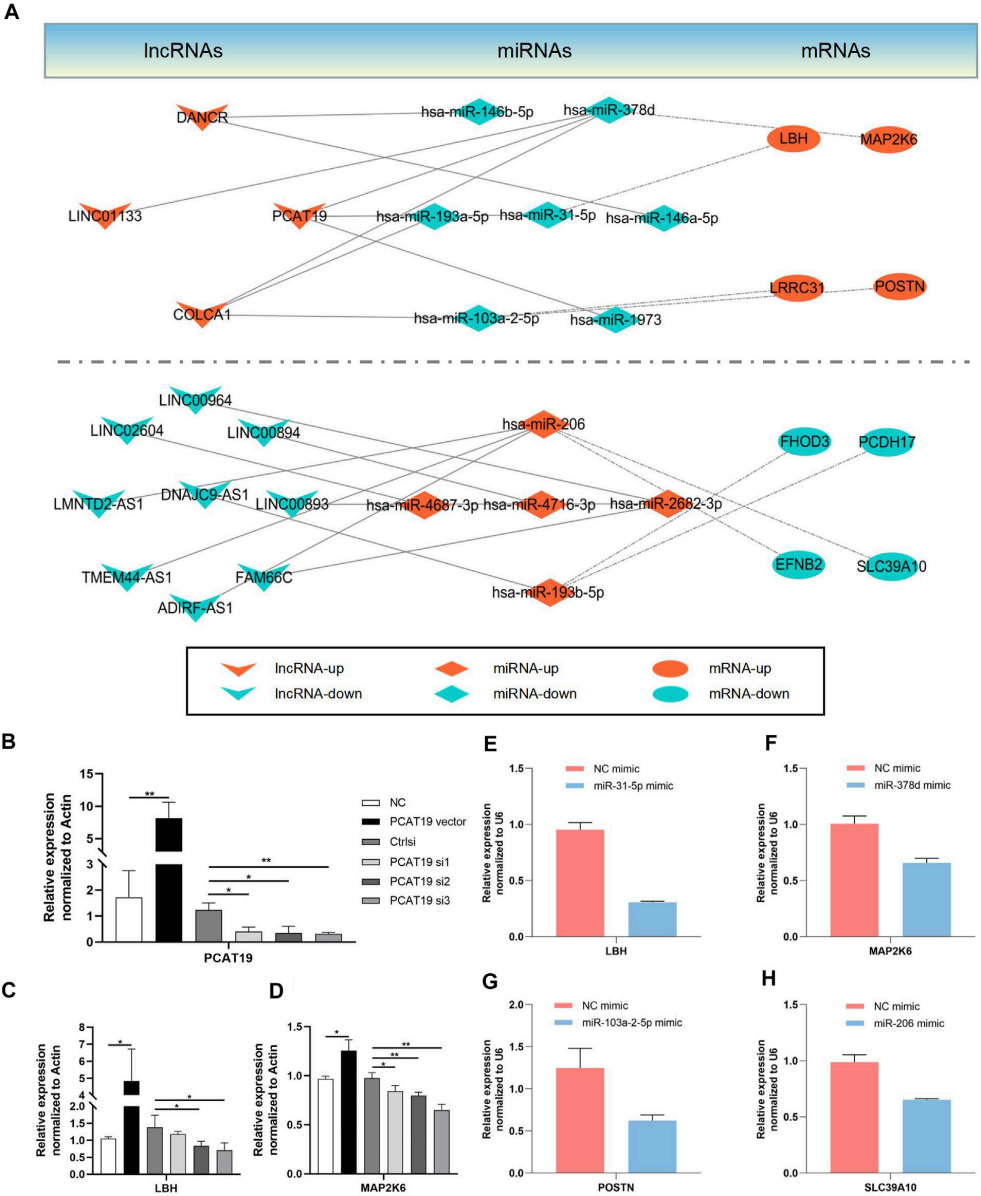
Establishment of a ceRNA network. The lncRNA-miRNA interactions are indicated by solid lines. The mRNA-miRNA interactions are indicated by dashed dot lines. Arrowhead represents lncRNAs; Rhombus represents miRNAs; Ellipse represents mRNAs. **(A)** DElncRNA-mediated ceRNAs network **(B)** Transfection and silencing efficiency of PCAT19 were determined concomitantly by qRT-PCR. **(C–H)** The representative candidate genes regulated in the ceRNA network were confirmed by qRT-PCR. *p < 0.05; **p < 0.01.

CeRNA has been found as a vital regulatory mechanism of lncRNA. To explore the correlation among the screened lncRNAs, miRNAs and mRNAs, a ceRNA network was constructed in accordance with their interactions. Moreover, the network was classified into two groups based on the up-down relationship; the results are presented in Figure 8A. Furthermore, the lncRNA, *PCAT19*, with the largest logFC in the bioinformatic analysis was adopted to verify the network’s reliability. A lncRNA *PCAT19*-overexpression vector and three small interfering RNAs (siRNAs) targeting different regions of lncRNA-*PCAT19* sequencing were established to assess the effect of PCAT19 expression on its downstream effector. The transfection and knockdown efficiencies in airway epithelial cell lines (16HBE) were validated through the qRT-PCR experiments. As depicted in Figure 8B, siRNA3-*PCAT19* most significantly inhibited the expression of *PCAT19* (*p* < 0.01). Since *LBH* and *MAP2K6* were downstream mRNAs of lncRNA *PCAT19* in the ceRNA network, the effects of an increase or knockdown of the expression of *PCAT19* were identified. Similar to the correlation in the ceRNA network, the up-regulation of *PCAT19* led to the increase in the expressions of the above two molecules (*LBH* and *MAP2K6)*, whereas knockdown down-regulated the expression without affecting corresponding miRNA (Figures 8C,D; Supplementary Figure S2).

In accordance with the ceRNA network hypothesis, miRNAs, in most cases, target mRNA and indirectly regulate their expression. Thus, 16HBE cells were transfected with the corresponding mimic-miRNA in the subsequent experiments. Four representative pairs of miRNA-mRNA were selected to confirm the interaction between miRNA and mRNA in the ceRNA network, and their respective targeted mRNA expression was detected (Figures 8E–H). Consistent with the hypothesis of this study, it was observed that the expressions of corresponding targeted genes in the miRNA overexpression group were down-regulated. The above results confirmed the reliability of the constructed ceRNA network.

## Discussion

Asthma refers to a heterogeneous disease characterized by chronic airway inflammation, airway remodeling, as well as airway hyperresponsiveness (Baggott and Beasley, 2020; Barcik et al., 2020). The increasing prevalence has exerted substantial medical and economic effects on individuals, society and the nation (Beasley and Hancox, 2020; Pijnenburg and Fleming, 2020). As mentioned above, asthma can fall into T2 and non-T2 types based on Th2 cytokines and their corresponding signaling pathways. To be specific, the former category, classical inflammatory asthma phenotype, accounts for most asthma cases. Thus, the immunopathogenesis should be clarified to find potential biomarkers, so as to facilitate guide treatment.

As high-throughput sequencing technology has been leaping forward, much more attention has been paid to the function of lncRNA over the past few years. According to existing studies, lncRNA has been found a non-coding gene and does not play a role in biological processes *in vivo*. However, in-depth research has proven that lncRNA plays significant roles in a wide variety of biological processes (e.g., transcriptional activation, transcriptional interference, intranuclear transport, as well as other regulatory functions) (Kopp and Mendell, 2018). Some reports have documented that lncRNA is involved in the pathogenesis of asthma. For instance, Zhang et al. (2021) suggested that lncRNA, *CRNDE,* can facilitate the proliferation and migration of airway smooth muscle cells in asthma. However, the above studies have been inadequate, especially in the field of *in vitro* studies. The lncRNA-miRNA-mRNA regulatory network has been found to play essential roles in various diseases (Jiang et al., 2020; Tang et al., 2021). In this study, a lncRNA-mediated ceRNA network for T2 asthma was established; the genes relating to T2 asthma were analyzed using the WGCNA method. To the best of our knowledge, this study has been the first to create a lncRNA-mediated ceRNA network for T2 asthma.

First, differentially expressed T2 asthma-related lncRNA and miRNA were acquired from GSE67472 and GSE142237. Their AUC values were obtained, and their inter-group differences were investigated using violin plots to prove the diagnostic value of the overlapping up-regulated DElncRNAs. IL-13 or IL-33 stimulation of 16 human bronchial epithelial (16HBE) cells was conducted to simulate the cell environment of T2 asthma. Subsequently, the consistency of the qRT-PCR and RNA sequencing results was observed. The correlation between lncRNA and miRNA were predicted based on the Diana-LncBase V2 database to build a lncRNA-miRNA network. Moreover, the expression genes of asthma patients and healthy controls with the top 40% variant values were selected for WGCNA network analysis, and the genes in the module relating to T2 asthma were selected. Furthermore, the differentially expressed mRNAs between asthma patients and healthy controls were investigated. The Th2-related genes analyzed by WGCNA were intersected with the differential genes between asthma patients and healthy controls. The overlapping genes were investigated in depth.

Furthermore, the overlapping genes were enriched and analyzed. The correlations between the miRNAs and overlapping mRNAs were predicted using targetscan 7.2, mirwalk 2.0, as well as miRDB. The lncRNA, miRNA and mRNA regulatory networks were constructed with intersecting genes. Moreover, the correlation between *PCAT19* and its downstream mRNAs in the ceRNA network was confirmed. Lastly, four representative miRNA-mRNA pairs were selected for an in-depth verification of the network’s credibility.

*PCAT19,* the lncRNA with the most significant difference in the bioinformatic analysis and the largest area under the ROC curve, was selected for in-depth investigations. *PCAT19* plays a role in the occurrence and development of various diseases, whereas its role in asthma—especially in the T2 asthma type—remains unclear. Hua et al. first reported *PCAT19* (Hua et al., 2018). Existing studies have suggested that it could interact with *HNRNPAB* to facilitate the progression and metastasis of prostate cancer. *PCAT19* is significantly down-regulated in non-small cell lung carcinoma and may be involved in its pathogenesis (Acha-Sagredo et al., 2020). Moreover, *PCAT19* can promote the proliferation of laryngeal cancer cells through the *miR-182/ PDK4* axis (Xu et al., 2019). In the non-tumor aspect, *PCAT19* has been found to play a role in the occurrence of neuropathic pain by targeting the *mir-182-5p*–*JMJD1A* axis (Huo et al., 2021). Presently, distinguishing between T2 asthma and non-T2 asthma is difficult. Accordingly, a biomarker that distinguishes the two types can provide a new direction for diagnosis and treatment. In addition, lncRNA is capable of competitively inhibiting the effect of miRNAs on downstream mRNA. Our study findings suggested that *PCAT19* might affect downstream *LBH* and *MA2PK6* by this effect. Accordingly, further experiments are required to investigate whether *PCAT19* plays a role in the pathogenesis of asthma through the above axes.

## Conclusion

In this study, the differential genes correlated with T2 asthma from the GEO online database were explored using a collection of bioinformatic methods. The lncRNA-mediated ceRNA regulatory networks in T2 asthma were built for the first time, which might provide new insights into diagnosis, pathogenesis and therapy. Moreover, the integrative analysis of this study confirmed the potential of *PCAT19* in the diagnosis and classification of asthma. Clarifying the pathophysiologic function of *PCAT19* in asthma may further increase its clinical significance.

## Data Availability

The datasets presented in this study can be found in online repositories. The names of the repository/repositories and accession number(s) can be found in the article/Supplementary Material.

## Abbreviations

BP, biological process; CC, cellular component; DEGs, differentially expressed genes; GEO, gene expression omnibus; GO, gene ontology; HBE, human bronchial epithelial; KEGG, Kyoto encyclopedia of genes and genomes LncRNA, long noncoding RNA; limma, linear models for microarray data; MF, molecular function; miRNA, microRNA; mRNA: messenger RNA; NCBI, national center for biotechnology information; PCA, principal component analysis; ROC, receiver operating characteristic; qRT-PCR, quantitative real-time polymerase chain reaction; siRNAs: small interfering RNAs; TOM, topological overlap matrix; WGCNA, weighted gene co-expression network analysis.
