# Supplementary figures and images for: Construction of lncRNA-Mediated Competing Endogenous RNA Networks Correlated With T2 Asthma
Source: Front Genet. 2022 Apr 11;13:872499. doi: 10.3389/fgene.2022.872499 (PMC9035528; doi:10.3389/fgene.2022.872499)

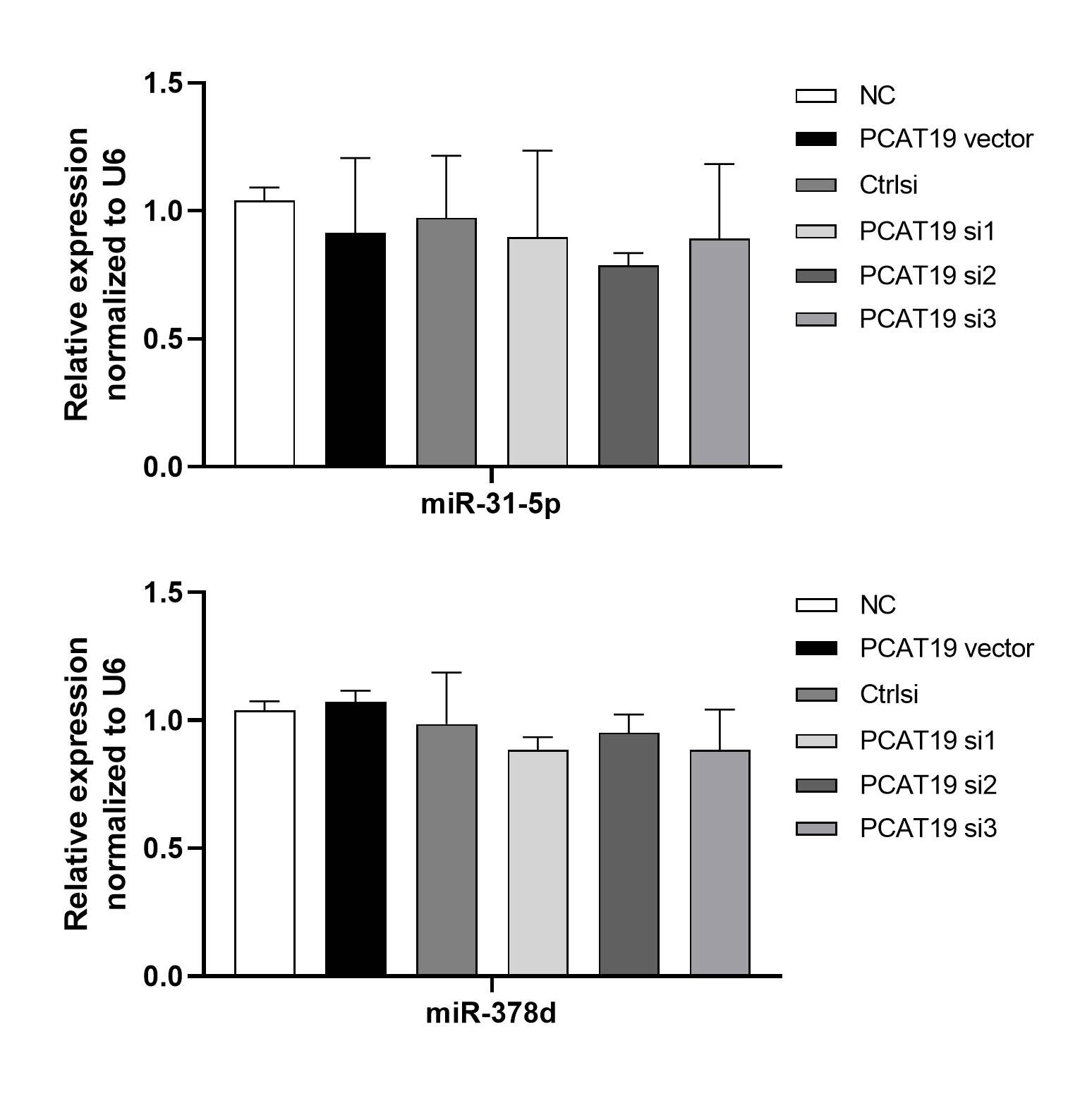

Supplement: Supplementary file 2 [file Image2.JPEG]
